# Adaptive Approach to Time-Frequency Analysis of AE Signals of Rocks

**DOI:** 10.3390/s22249798

**Published:** 2022-12-13

**Authors:** Olga Lukovenkova, Yuri Marapulets, Alexandra Solodchuk

**Affiliations:** Laboratory of Acoustic Research, Institute of Cosmophysical Research and Radio Wave Propagation FEB RAS, Kamchatka Region, Elizovskiy District, Mirnaya Str. 7, 684034 Paratunka, Russia

**Keywords:** acoustic emission, combined receiver, time-frequency analysis, adaptive matching pursuit, 43.58.Gn, 43.60.Mn

## Abstract

The paper describes a new adaptive approach to the investigation of acoustic emission of rocks, the anomalies of which may serve as short-term precursors of strong earthquakes. The basis of the approach is complex methods for monitoring acoustic emission and for analysis of its time-frequency content. Piezoceramic hydrophones and vector receivers, installed at the bottom of natural and artificial water bodies, as well as in boreholes with water, are used as acoustic emission sensors. To perform a time-frequency analysis of geoacoustic signals, we use a sparse approximation based on the developed Adaptive Matching Pursuit algorithm. The application of this algorithm in the analysis makes it possible to adapt to the concrete characteristics of each geoacoustic pulse. Results of the application of the developed approach for the investigation of acoustic emission anomalies, occurring before earthquakes, are presented. We analyzed the earthquakes, that occurred from 2011 to 2016 in the seismically active region of the Kamchatka peninsula, which is a part of the circum-Pacific orogenic belt also known as the “Ring of Fire”. It was discovered that geoacoustic pulse frequency content changes before a seismic event and returns to the initial state after an earthquake. That allows us to make a conclusion on the transformation of acoustic emission source scales before earthquakes. The obtained results may be useful for the development of the systems for environmental monitoring and detection of earthquake occurrences.

## 1. Introduction

One of the important problems in geophysics is the detection, analysis, and explanation of the reasons for the anomalies, which are observed before earthquakes. These anomalies are found in the data of different physical fields and can be interpreted as earthquake precursors. One of such pre-seismic anomalies is the increase in intensity of acoustic emission (AE) of rocks in the frequency range from hundreds of hertz to the first tens of kilohertz. It is observed in different seismically active regions of the world such as Armenia [1], Italy [2], Romania [3], and Russia [4].

**Figure 1:**
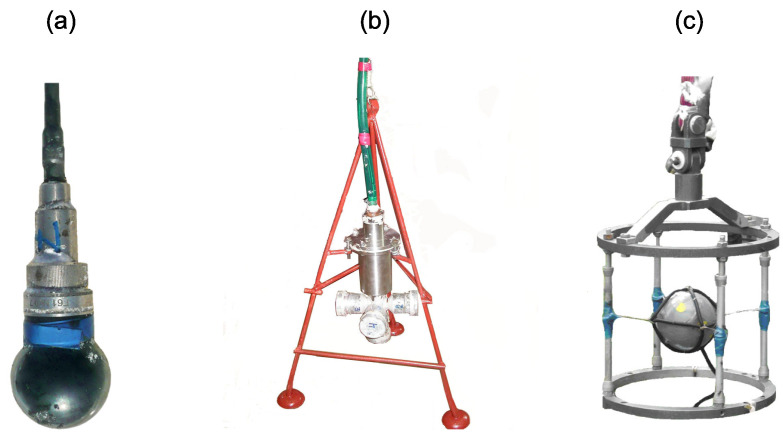
AE sensors: (**a**) hydrophone with integrated amplifier, (**b**) system of directed hydrophones, (**c**) combined receiver.

One of the most seismically active regions of Russia is the Kamchatka Peninsula, which is a part of the circum-Pacific orogenic belt, also known as the “Ring of Fire”. In the result of long-term investigations of AE in the seismically active region of the Kamchatka peninsula, we detected a high-frequency acoustic emission effect, which consists in the growth of geoacoustic emission intensity during the increase in deformation rate of rocks [5]. This effect is determined by deformations of rocks at observation sites and manifests the most vividly in the kilohertz frequency range 1–3 days before earthquakes at the distance of first hundreds of kilometers from their epicenters [6]. The peculiarity of the experiments in Kamchatka is the application of wide-band piezoceramic hydrophones, installed at the bottom of natural and artificial water bodies, to monitor AE. Application of such a type of receiver allows us to increase the upper boundary of the frequency range from 1–2 kHz to 10–20 kHz compared to standard geophones [7]. The emission signal, generated in the ground, is received in the water. As long as transverse waves do not propagate in a fluid, the sensors record only the longitudinal component of an acoustic wave. It is refracted at the boundary of two mediums. In this case, the refraction coefficient is about 1.2–1.7. Taking into account small distances of signal propagation (first tens of meters), the refraction effects can be neglected [8]. The results of experimental investigations in closed inner water bodies [4] and on the ocean shelve [9] show that at large distances, distortion of pulse signal forms is insignificant when they propagate in a waveguide consisting of a water layer and a near-surface ground layer. Thus, hydrophones, installed at the bottom of water bodies, can be used to record geoacoustic signals. To monitor AE in Kamchatka, we use hydrophones, which sensitivity together with preliminary amplifiers is of the order of tens-hundreds of millivolts per pascal (Figure 1a). Also, a system of four differently directed hydrophones was used (Figure 1b) [4]. To study the spatial structure of AE and medium particle motion character, a combined receiver was applied [8]. It consisted of a three-component vertical receiver and a hydrophone (Figure 1c).

It is known that AE in solid bodies is elastic oscillations occurring as the result of the local dynamic reconstruction of its structure. The main AE sources are fracture formation and extension in brittle bodies as well as dislocations in bodies having more ductile properties. Thus, the emitted oscillation characteristics are associated directly with the peculiarities of the solid body deformation process. Analysis of AE data of Kamchatka shows that the recorded signal consists of pulses of different amplitude and duration with shock excitation and the filling frequency from hundreds of hertz to tens of kilohertz [8]. The radiation intensity is determined by the deformation of rocks and increases before earthquakes.

To determine the main frequencies of AE during such pre-seismic anomalies, we divided the received signal into frequency sub-bands and analyzed the spectrum using the Fourier transform. It was discovered that the emission maximum before an earthquake is recorded in kilohertz frequency range [6]. It was difficult to make a more detailed analysis of the signal spectrum as long as the recorded AE pulse signals were characterized by a wide diversity of their waveforms and short duration [10]. Thus, the application of time-frequency analysis methods, used to solve such problems in allied sciences (Short Time Fourier transform, wavelet transform, wavelet packets, etc.), was of low efficiency.

The emission maximum in the kilohertz frequency range should indicate the change in AE source scales. Investigation of this process is very important for understanding the nature of the recorded pre-seismic AE anomalies. The relation between the source scale and emission frequency is confirmed by the known dislocation model by J. Brown

(1) $$l=2.34V_{p}/2\pi f,$$

where *l* is the source length, $V_{p}$ is the longitudinal oscillation velocity in rocks, *f* is the emission frequency.

As long as $V_{p}$ is constant for a certain region of observation, there is an inverse dependence between the source length and the radiation frequency. Rock strength in relation to shear stress is less than that of compression. That means that shear (dislocation) sources of acoustic emission prevail. Thus, it is acceptable to use this formula to estimate AE source scales.

To perform the time-frequency analysis of AE pulse signals, we suggested using the sparse approximation based on the Adaptive Matching Pursuit algorithm. That made it possible to adapt to concrete characteristics of each recorded pulse when analyzing emission frequency [10].

The paper describes a new approach to the investigation of AE characteristics. It is based on complex methods of monitoring AE signals and analysis of their time-frequency content by the Adaptive Matching Pursuit. For the first time ever, we present the results of an investigation of AE pulse frequency distribution before some earthquakes occurred in Kamchatka in 2011–2016. To do that, AE pulse frequencies were analyzed at different time intervals before and after earthquakes based on the developed approach.

## 2. Adaptive Matching Pursuit

Sparse approximation assumes searching for a linear equation system solution having the maximum possible sparseness (most parts of unknowns is equal to 0).

Sparse approximation ideas became a widespread practice in the processing of signals and images in different fields of science [11,12,13,14,15,16]. The sparse approximation works for nonstationary signal analysis better than classical processing methods such as Short Time Fourier Transform and wavelet transform [17].

The problem of searching for an exact solution of sparse representation is NP-difficult. However, there are methods allowing one to estimate an exact solution with some error over polynomial time. One of them is the Matching Pursuit, which is a greedy method bounding the number of non-zero unknowns above [17].

(2) $$\left\{\begin{array}{c} x\left(t\right)=\sum_{i=0}^{N-1}a_{i}g_{i}\left(t\right)+R\left(t\right)\\ ∥R(t)∥\to \min\\ {∥a∥}_{0}\to \min \end{array}\right.,$$

where x(t) is the signal; *N* is the number of functions, into which the signal x(t) is decomposed; $g_{i}\left(t\right)$ are the functions into which a signal is decomposed, $a_{i}$ are the decomposition coefficients; R(t) is the residual signal, ∥R(t)∥ is the $l_{2}$-norm; ${∥a∥}_{0}=\#\left\{i:\;a_{i}\neq 0,\;i\;=0\ldots N-1\right\}$ is the $l_{0}$-pseudonorm equal to the number of vector non-zero elements.

Matching Pursuit method is an iterative procedure of the following form

(3) $$\begin{array}{c} k=\arg\left[\max_{0\leq j<N}\left|\langle g_{j}\left(t\right),R_{i}\left(t\right)\rangle \right|\right],\\ R_{i+1}\left(t\right)=R_{i}\left(t\right)-\langle g_{k}\left(t\right),R_{i}\left(t\right)\rangle g_{k}\left(t\right). \end{array}$$

Signal x(t) is used as the initial value $R_{0}\left(t\right)$.

The following terminology was introduced by S. Mallat who developed the Matching Pursuit [17]. The function $g_{i}\left(t\right)$ is called “time-frequency atoms” and a set of these functions is a “dictionary”. The convergence accuracy and the rate of decomposition (2) depends on the selected dictionary. If as $g_{i}\left(t\right)$ we choose the functions constructed by scaling, shift, and frequency modulation of time-localized function g(t), then decomposition (2) for N→∞ converges to x(t) [17]. Thus, function $g_{i}\left(t\right)$ takes the form

(4) $$g_{i}\left(t\right)=\frac{1}{\sqrt{s_{i}}}g\left(\frac{t-\tau_{i}}{s_{i}}\right)\exp\left(j\omega_{i}t\right),$$

where $\tau_{i}$ is the shift, $s_{i}$ is the scale, $\omega_{i}$ is the frequency (thus, *i* uniquely corresponds to the triple $\tau_{i},s_{i},\omega_{i}$).

Best results can be obtained when the form of $g_{i}\left(t\right)$ corresponds to the initial signal x(t).

In seismology, models suggested by N. N. Puzyrev (Gaussian functions), and G. P. Berlage are often used for the analytical description of seismic-acoustic oscillations [18]. Taking into account the known property of spectral-time self-similarity of acoustic emission signals, we considered it reasonable to use these functions for sparse approximation of higher-frequency geoacoustic signals [19].

We used the following analytical representation of the modulated Gaussian function

(5) $$g\left(t\right)=A\exp\left(-B_{\lim}\left(T_{\mathrm{end}}\right)\Delta t^{2}\right)\cos\left(2\pi ft\right),\;-T_{\mathrm{end}}/2\leq t\leq T_{\mathrm{end}}/2,$$

where *A* is the amplitude selected so that ∥g(t)∥=1; $T_{\mathrm{end}}$ is the length of function domain interval; $B_{\lim}\left(T_{\mathrm{end}}\right)$ is the limit of the parameter *B*, estimated so that pulse amplitude on domain boundaries is 5% from the maximum value, $g\left(\pm T_{\mathrm{end}}/2\right)=0.05g\left(0\right)$; Δ is the coefficient of the parameter *B* variability relatively the limit value, Δ≤1, the higher Δ value is, the faster the tails of g(t) attenuate; *f* is the frequency (Hz). Similar to the Gaussian function, the following expression describes the Berlage function

(6) $$g\left(t\right)=At^{n_{\lim}\left(p_{\max}\right)\Delta }\exp\left(-\frac{n_{\lim}\left(p_{\max}\right)\Delta }{p_{\max}T_{end}}t\right)\sin\left(2\pi ft\right),\;0\leq t\leq T_{\mathrm{end}},$$

where *A* is the amplitude chosen so that ∥g(t)∥=1; $T_{\mathrm{end}}$ is the length of function domain interval; $p_{\max}$ is the parameter which sets the position of maximum relative to $T_{\mathrm{end}}$, $t_{\max}=p_{\max}T_{\mathrm{end}}$; $n_{\lim}\left(p_{\max}\right)$ is the parameter *n* limit estimated so that pulse amplitude at the domain boundary is 5% from the maximum value, $g\left(T_{\mathrm{end}}\right)=0.05g\left(t_{\max}\right)$; Δ is the coefficient of parameter *n* variability relatively the limit value, Δ≤1, the higher Δ value is, the faster the tail of g(t) attenuates; *f* is the frequency (Hz).

**Figure 2:**
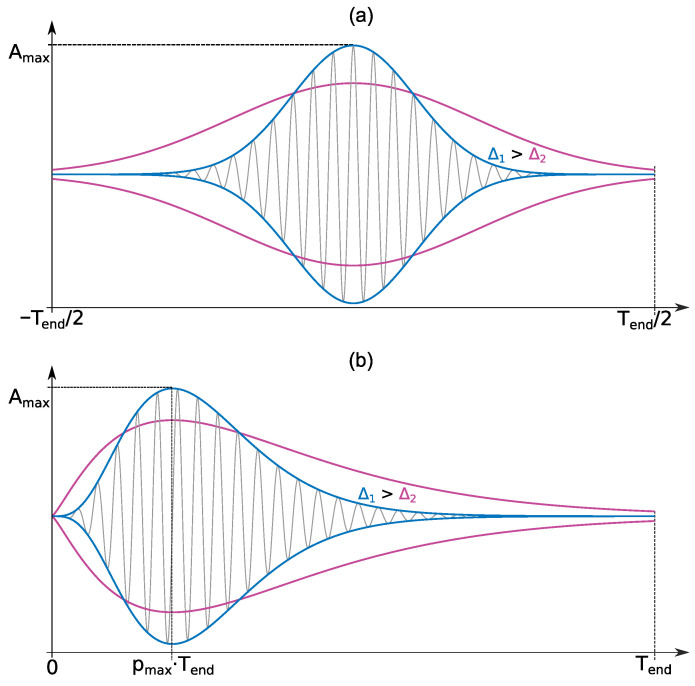
Effect of the parameters $T_{\mathrm{end}}$, $p_{\max}$ and Δ on the form of functions: (**a**) Gaussian function, (**b**) Berlage function.

The effect of the parameters $T_{\mathrm{end}}$, $p_{\max}$ and Δ on the form of functions is shown in Figure 2, where the red line corresponds to a lower value of the parameter Δ, and the blue line corresponds to a higher one.

Value ranges were experimentally picked out for each parameter $T_{\mathrm{end}}$, $p_{\max}$, Δ and *f* from the suggested dictionaries (5) and (6). They made it possible to approximate geoacoustic signals with high accuracy.

We improved classical Matching Pursuit algorithm (3). Matching Pursuit execution time depends on the cardinality of a dictionary applied ($O(N^{2}\log N)$), and it takes dictionaries of large volumes to obtain sparse representations of high accuracy. To solve this problem we added updating procedure of the parameters $p=T_{\mathrm{end}},\Delta ,f$ (for the Gaussian function) or $p=T_{\mathrm{end}},p_{\max},\Delta ,f$ (for the Berlage function) of the atom determined at each iteration

(7) $$F\left(\tau ,p\right)=\left|\langle R\left(t\right),g\left(t-\tau ,p\right)\rangle \right|\to \max_{p},$$

where τ is the time shift of the function, all possible shifts relative to the initial signal are taken into account.

Optimization (7) can be done by different methods, for example, by the method of gradient descent and grid search. Computational experiments showed that the best result is achieved when using the grid search method separately for the frequency *f* and the parameters affecting the envelope form ($T_{\mathrm{end}}$, Δ for the Gaussian function and $T_{\mathrm{end}}$, $p_{\max}$, Δ for the Berlage function). The modified algorithm of Matching Pursuit with the updating procedure was called Adaptive Matching Pursuit [20]. The algorithm can be described as follows.

1. $R_{0}\left(t\right)=x\left(t\right)$, i=0.
2. Define atom $g_{k}\left(t,p_{0}\right)$ from the decomposition (2) by classical Matching Pursuit (3), j=0.
3. Set grid step for each parameter as half of the sampling interval of this parameter $\lambda =\{0.5\lambda_{\max},0.5\lambda_{\Delta },0.5\lambda_{\mathrm{end}},0.5\lambda_{f}\}$.
4. In the neighborhood of $p_{j}$, construct a new grid, containing three points for each parameter *p*: p−λ, p, p+λ.
5. Define atom with the biggest correlation with $R_{i}\left(t\right)$ among the atoms corresponding to the grid nodes. $p_{j+1}$ is the atom parameters.
6. If λ is less than a minimum possible step (corresponds to selected accuracy) then go to step 9.
7. If $p_{j+1}=p_{j}$ then decrease the step λ=λ/2.
8. j=j+1, repeat from step 4.
9. Estimate the residual signal $R_{i+1}\left(t\right)$ using $g_{k}\left(t,p_{j}\right)$.
10. If the ratio of the residual signal norm to the initial signal norm becomes less than the prescribed threshold ε ($∥R_{i}\left(t\right)∥/∥x\left(t\right)∥<\varepsilon$) then STOP, otherwise i=i+1 and repeat from step 2.

Owing to the updating procedure, it is possible to obtain sparse approximations of the same accuracy in the dictionaries of less cardinality [10].

## 3. Description of the Experiment

**Figure 3:**
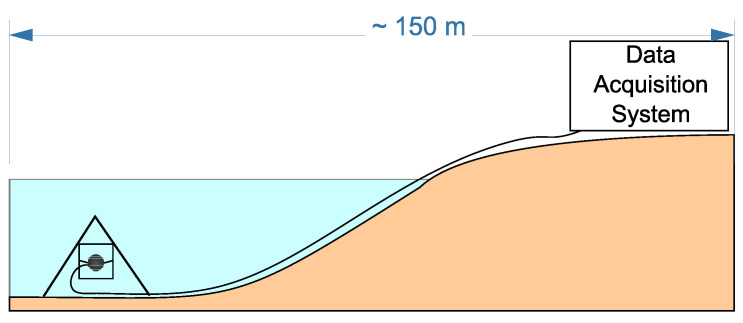
Scheme of the combined receiver setup at the bottom of Mikizha lake.

To record acoustic emission signals, a combined receiver was used. It consists of a three-component vector receiver and a hydrophone (Figure 1c) and is installed at the bottom of Mikizha lake (52.99${}^{\circ }$ N, 158.23${}^{\circ }$ E) in the seismically active region of the Kamchatka peninsula. This lake is located in the region of a tectonic fracture. Cases of anomalous pre-seismic disturbances of acoustic emission had been multiply recorded at this point before [4,5,6]. A scheme of the receiver setup is illustrated in Figure 3. This paper analyzes the data obtained from the vertically oriented channel of the vector receiver (ground-surface direction) because the signal from this channel is less noisy. Original data collected by the Laboratory of Acoustic Research of IKIR FEB RAS for the period 2011–2016 were under the analysis.

The signal was recorded in the range of 20 Hz–20 kHz, digitized, and written on a PC hard disk. The data were stored as sequential 15-min files in audio format. Time-frequency processing was realized by using video card processors that made it possible to parallelize calculation operations and to realize signal analysis in near real-time mode. The data were thoroughly pre-processed. As the result, the audio files, containing meteorological and industrial noises, were removed. After that, pulses were distinguished in each audio file by an original adaptive threshold algorithm [21].

**Figure 4:**
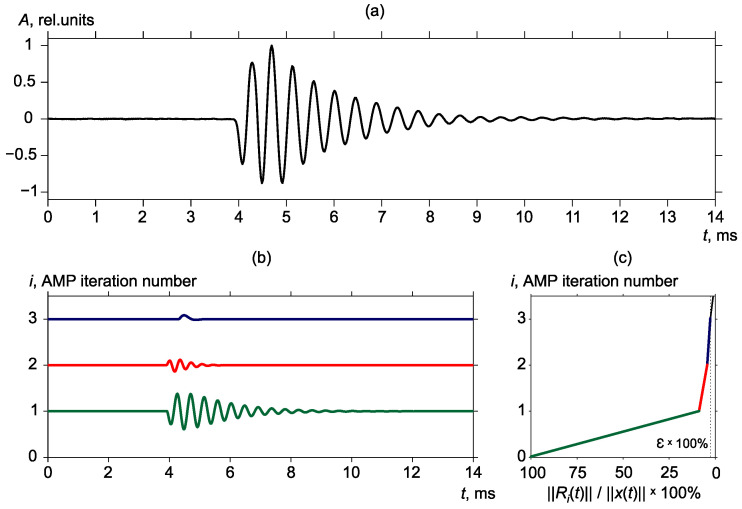
Example of geoacoustic pulse decomposition. (**a**) Geoacoustic pulse, (**b**) the first three atoms (each atom is marked by a different color) included into pulse decomposition, (**c**) graph of value $∥R_{i}\left(t\right)∥/∥x\left(t\right)∥$ fall.

Then for each detected pulse, a sparse representation was constructed by the Adaptive Matching Pursuit algorithm. As the result, each detected pulse was represented in the form of a combination of the minimally possible number of shifted and modulated (from 10 Hz to half of the sampling frequency) Gaussian and Berlage functions (2). Each function is included in decomposition with a certain coefficient; the most powerful frequency of a pulse is described by the function with the highest modulo coefficient (Figure 4). Analysis of a large number of geoacoustic signals, recorded in Kamchatka, shows that this function amplitude usually exceeds the amplitudes of the rest of atoms from decomposition. However, the frequency characteristics of a pulse, taken by itself, is less informative since recorded signals consist of a great number of geoacoustic pulses generated by a large number of sources. That is why we further analyzed the changes in the distributions of the most powerful frequencies of pulses at finite time intervals of prescribed duration. In this paper, a 1-day interval was used. This interval was chosen based on the results of the analysis of a large volume of acoustic emission data as long as in this interval the changes of signal characteristics are most clearly manifested.

The results of the time-frequency analysis were compared with the earthquakes that occurred from 2011 to 2016 near the Kamchatka peninsula. Earthquakes with local magnitude $M_{L}\geq 4.7$ (536 in total) were selected from the seismic catalog for Kamchatka and Komandorskie Islands [22]. To estimate the radius $R_{D}$ of the regions around a preparing earthquake, where anomalies in geophysical fields of different nature can be observed, the following formula was proposed [23]

$$R_{D}=10^{0.43M_{W}},$$

where $M_{W}$ is the moment earthquake magnitude. In this paper, we use the local magnitude $M_{L}$. For Kamchatka earthquakes, the relationship between $M_{W}$ and $M_{L}$ is $M_{W}=M_{L}+0.4$ [24].

Investigation results show that this expression agrees well with real distances to the epicenters of Kamchatka earthquakes, before which anomalies in rock AE were recorded [25,26]. The selected earthquakes were divided into three groups depending on the distance between the earthquake epicenter and the hydrophone location. The first group included the earthquakes, the epicentral distances of which were up to one $R_{D}$ (101 earthquakes). The second group included the earthquakes, the epicentral distances of which were from one $R_{D}$ to two $R_{D}$ (196 earthquakes). The rest of the earthquakes were referred to as the third group.

In the work, we analyzed distributions of the most powerful frequencies of geoacoustic pulses recorded before and after the earthquakes from the first and the second groups on the intervals ±10 days relatively earthquake time. Estimates of frequency distribution probability density were plotted for these intervals. To do that, a histogram method (interval length is 500 Hz) and a kernel density estimate method were used [27,28]. Earthquakes of the third group were not analyzed as long as their epicenters were far away from the observation site.

**Figure 5:**
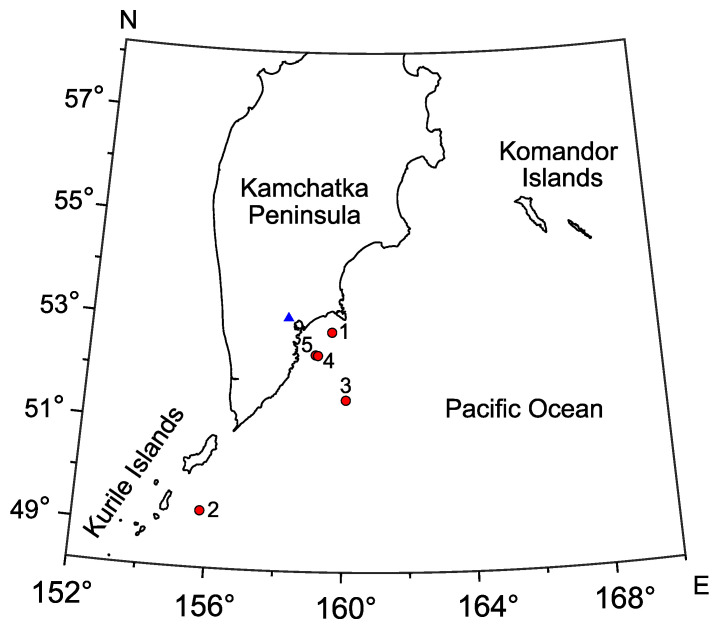
Kamchatka peninsula map with locations of epicenters of earthquakes considered in the paper. Blue triangle (▴) indicates Mikizha lake, red circles (•) are earthquake epicenters.

Hereinafter we present the most interesting cases of distribution change for the most powerful pulse frequencies during earthquake preparation and development. Earthquake characteristics are listed in Table 1. Earthquake epicenters are shown on the map (Figure 5). Earthquake numbers on the map correspond to the numbers in Table 1. Earthquakes are enumerated according to the order they are considered in the paper.

**Figure 6:**
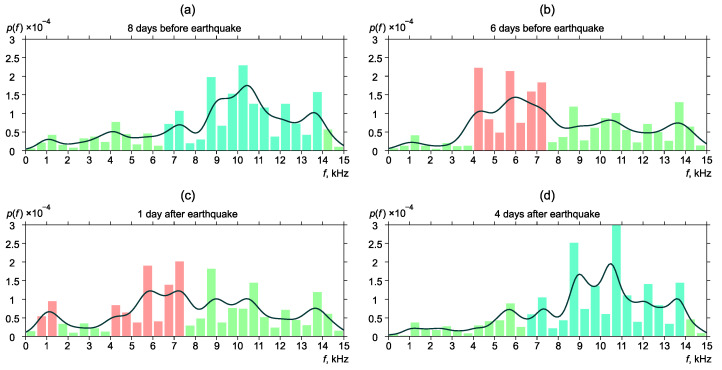
Dynamics of the probability density of distribution of the most powerful frequencies of geoacoustic pulses before and after the earthquake No. 1. p(f) is the frequency density.

**Figure 7:**
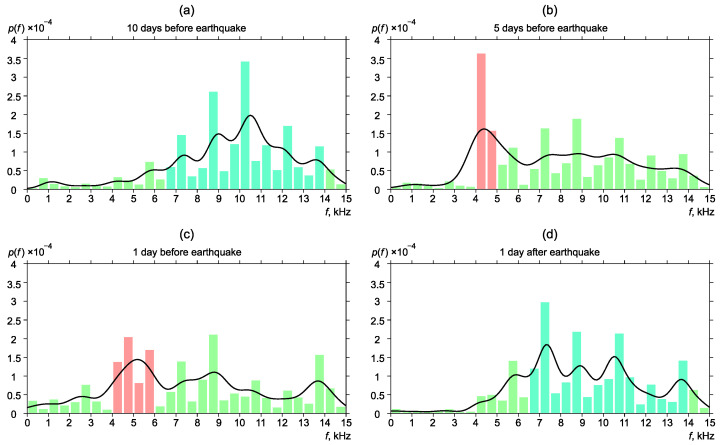
Dynamics of the probability density of distribution of the most powerful frequencies of geoacoustic pulses before and after the earthquake No. 2.

**Figure 8:**
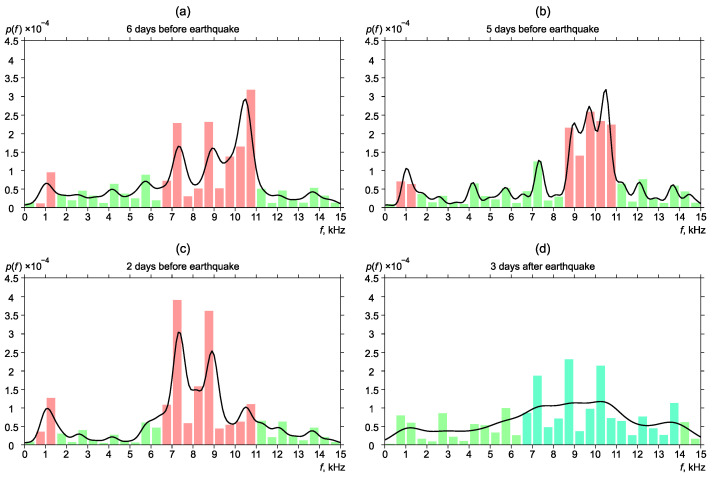
Dynamics of the probability density of distribution of the most powerful frequencies of geoacoustic pulses before and after the earthquake No. 3.

**Figure 9:**
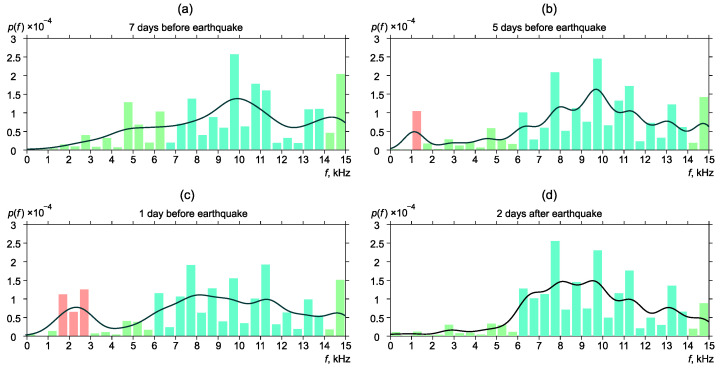
Dynamics of the probability density of distribution of the most powerful frequencies of geoacoustic pulses before and after the earthquake No. 4.

**Figure 10:**
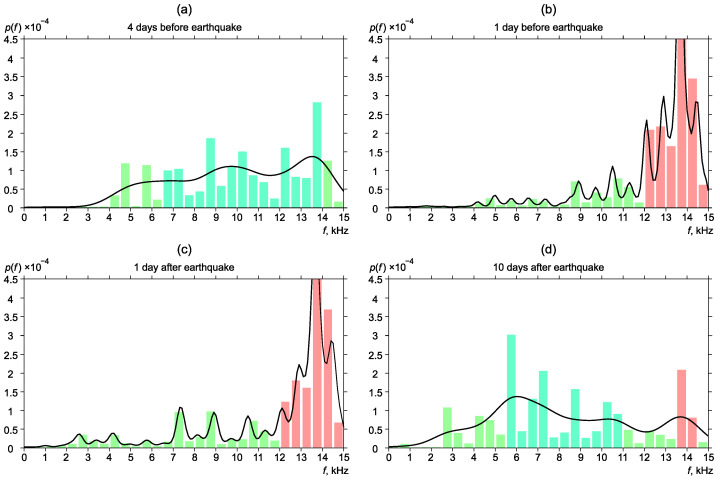
Dynamics of the probability density of distribution of the most powerful frequencies of geoacoustic pulses before and after the earthquake No. 5.

Figure 6, Figure 7, Figure 8, Figure 9 and Figure 10 show the changes of frequency distribution probability density before and after the earthquakes listed in Table 1. The bar graph illustrates the estimate obtained by the histogram method. The line shows the kernel density estimate of probability density. Blue bars show the frequency range in which emission maxima are recorded during seismically quiet periods. Red bars show anomalies in the frequency distribution.

Eight days before the earthquake No. 1, the most powerful frequencies of geoacoustic pulses were mainly focused within the range of 6.5–14 kHz (Figure 6a). We considered such a distribution to be “background” as long as similar estimates of the probability density of frequency distribution are typical for the most part of the data analyzed. Six days before the earthquake, distribution maximum shift to the region of 4–7.5 kHz was observed (Figure 6b). On the day, when the earthquake occurred, the pick remained in the region of 4–7.5 kHz. We can also note the increase of the pulse segment with the frequency of 0.5–1.5 kHz (Figure 6c). Frequency distribution returned to the background four days after the earthquake (Figure 6d).

A similar distribution of frequencies was observed in the case of earthquake No. 2. Figure 7 illustrates the changes in frequency distribution probability density for this case.

The figure (Figure 7a) shows that most of the pulses had a frequency from the range of 6.5–14 kHz (background distribution) ten days before the earthquake. Five days before the earthquake, a pick shift to the region of 4–5 kHz was observed (Figure 7b). One day before the earthquake, frequency distribution began to return to the background. The maximum was smoothly moving to the higher-frequency region 4–6 kHz (Figure 7c). One day after the earthquake, frequency distribution returned to the background form (Figure 7d).

One more example of distribution change of the most powerful frequencies near the earthquake No. 3 is illustrated in Figure 8.

Six days before the earthquake, the frequencies were focused on the region of 6.5–11 kHz. Also, a weak pick was observed in the region of 0.5–1.5 kHz (Figure 8a). Five days before the earthquake, an increase of the pulse segment with the frequency of 0.5–1.5 kHz was observed (Figure 8b). A similar picture was observed two days before the earthquake (Figure 8c). The distribution returned to the background form gradually (Figure 8d).

Similar changes in frequency distribution were observed before and after earthquake No. 4 (Figure 9). Seven days before the earthquake, frequency distribution was background one. The distribution probability density pick was in the frequency range of 6.5–14 kHz (Figure 9a). Five days before the earthquake, an increase of the pulse segment with the frequencies corresponding to the region of 1–1.5 kHz (Figure 9b) was observed. During the day, the pick moved to the region of 1.5–3 kHz (Figure 9c). Two days after the earthquake, frequency distribution returned to the background form (Figure 9d).

One more interesting case of frequency distribution change was detected before earthquake No. 5 (Figure 10). Unlike the cases described above, the maximum distribution of the most powerful frequencies shifted to the higher-frequency region. Four days before the earthquake, frequency distribution corresponding to the case of background distribution (Figure 10a). One day before the earthquake, the segment of high-frequency pulses (12–15 kHz) suddenly increased (Figure 10b). The situation remained one day after the earthquake (Figure 10c). Ten days after the earthquake, frequency distribution recovered to the background form (Figure 10d).

## 4. Conclusions

A new approach to the investigation of AE of rocks, which anomalies may serve as short-term precursors of earthquakes, is suggested. Piezoceramic hydrophones and vector receivers are used as AE sensors. They are installed at the bottom of natural and artificial water bodies. To perform time-frequency analysis of geoacoustic pulse signals, a sparse approximation based on the Adaptive Matching Pursuit is used. The application of this algorithm makes it possible to adapt to the concrete characteristics of each geoacoustic pulse during the analysis. Realization of the time-frequency processing on video card multicore processors allows us to parallelize computation operations and to perform signal analysis in near real-time mode.

To detect AE anomalies associated with earthquakes, data on the intervals of ±10 days relative earthquake times were analyzed on the basis of the suggested approach. Earthquakes, that occurred near the Kamchatka peninsula during the period 2011–2016, were considered. It was determined during the analysis that changes in the frequency content of geoacoustic pulses occur before seismic events. Redistribution of the most powerful frequencies, compared to the background regime, takes place. That is followed by distribution return to the initial form after an earthquake.

The most interesting cases of such changes are presented. The detected changes in signal frequency content allow us to make the conclusion that the scales of AE sources change as long as the pulse frequency is determined by the source scale.

Thus, the application of the developed method to monitor AE pulse signals and to analyze their time-frequency content makes it possible to detect changes in their frequency content in near real-time mode. The method may be the base for the development of the systems for environmental monitoring and detection of earthquake occurrences.

## Figures and Tables

**Table 1:** Earthquake characteristics.

No.	Epicenter Coordinates	Date and Time, UT	Local Magnitude $M_{L}$	Depth, km	Epicentral Distance *R*, km	Earthquake Group
1	52.84${}^{\circ }$ N, 159.62${}^{\circ }$ E	2012.09.24 14:39:52.9	4.8	65	95	1
2	49.30${}^{\circ }$ N, 155.78${}^{\circ }$ E	2012.11.01 06:57:19.7	5.5	76	444	2
3	51.53${}^{\circ }$ N, 160.08${}^{\circ }$ E	2012.10.15 01:18:58.6	6.0	44	205	1
4	52.38${}^{\circ }$ N, 159.22${}^{\circ }$ E	2015.12.16 08:20:15.5	5.0	72	95	1
5	52.40${}^{\circ }$ N, 159.08${}^{\circ }$ E	2013.01.19 16:48:09.2	4.9	56	87	1

## Data Availability

The data on earthquakes considered in the article are available at Unified Information System of Seismological Data of the Kamchatka Branch of Geophysical Service Russian Academy of Science at http://sdis.emsd.ru/info/earthquakes/catalogue.php (accessed on 22 November 2022).
